# Minimal hepatic encephalopathy is associated to alterations in eye movements

**DOI:** 10.1038/s41598-022-21230-3

**Published:** 2022-10-07

**Authors:** Franc Casanova-Ferrer, Cecilia E. García-Cena, Juan-Jose Gallego, Alessandra Fiorillo, Amparo Urios, Alberto Calvo-Córdoba, Maria-Pilar Ballester, María Pilar Ríos, Lucía Durbán, Marta R. Hidalgo, Francisco García, Vicente Felipo, Carmina Montoliu

**Affiliations:** 1 Fundación de Investigación Hospital Clínico Universitario de Valencia-INCLIVA, 46010 Valencia, Spain; 2 Centre for Automation and Robotics, Universidad Politecnica de Madrid, Madrid, Spain; 3 Servicio de Medicina Digestiva, Hospital Clínico Universitario de Valencia, 46010 Valencia, Spain; 4 Servicio de Medicina Digestiva, Hospital Arnau de Vilanova, 46015 Valencia, Spain; 5 Bioinformatics and Biostatistics Unit, Centro Investigación Príncipe Felipe, 46012 Valencia, Spain; 6 Laboratory of Neurobiology, Centro Investigación Príncipe Felipe, 46012 Valencia, Spain; 7 Department of Pathology, Faculty of Medicine, University of Valencia/INCLIVA-Health Research Institute, Avda. Blasco Ibañez, 17, 46010 Valencia, Spain

**Keywords:** Hepatology, Cognitive neuroscience, Neuroscience

## Abstract

Minimal hepatic encephalopathy (MHE) is diagnosed using PHES battery, but other tests are more sensitive, and a simple tool for early MHE detection is required. Assessment of saccadic eye movements is useful for early detection of cognitive alterations in different pathologies. We characterized the alterations in saccadic eye movements in MHE patients, its relationship with cognitive alterations and its utility for MHE diagnosis. One-hundred and eighteen cirrhotic patients (86 without and 32 with MHE) and 35 controls performed PHES and Stroop test and an eye movements test battery by OSCANN system: visual saccades, antisaccades, memory-guided saccades, fixation test and smooth pursuit. We analyzed 177 parameters of eye movements, assessed their diagnostic capacity for MHE, and correlated with cognitive alterations. MHE patients showed alterations in 56 of the 177 variables of eye movements compared to NMHE patients. MHE patients showed longer latencies and worse performance in most eye movements tests, which correlated with mental processing speed and attention impairments. The best correlations found were for antisaccades and memory-guided saccades, and some parameters in these tests could be useful for discriminating MHE and NMHE patients. Eye movements analysis could be a new, rapid, reliable, objective, and reproducible tool for early diagnose MHE.

## Introduction

Between 33 and 50% of patients with liver cirrhosis can develop minimal hepatic encephalopathy (MHE), the earliest form of hepatic encephalopathy (HE)^1–4^, which affects several million patients around the world^5^. MHE is characterized by mild cognitive impairment, alterations in attention^4,6,7^, psychomotor slowing and impaired motor coordination^7,8^, altered postural control^9^ associated with increased risk of falls^10^, and impaired fitness to drive^11,12^. MHE reduces life quality^13^, and increases the risk of progression to overt hepatic encephalopathy^14^.

Early detection and treatment of MHE would prevent its progression to HE, reduce hospitalization costs^15^, prolong survival^16,17^, and improve quality of life of patients.

There is a consensus to use the Psychometric Hepatic Encephalopathy Score (PHES) as a common “gold standard” for diagnosis of MHE^18,19^. The PHES is a battery of five psychometric tests (see Methods) that evaluates mainly mental processing speed, motor speed, attention, and visuo-spatial coordination^18^.

However, these tests are time consuming, require specialized staff, and must be corrected by age and educational level. Therefore, these tests are difficult to perform in clinical practice, and most patients with MHE remain undiagnosed and untreated around the world. There is a clear necessity to find new tools for early detection of MHE in an objective, rapid, reproducible and sensitive way.

Alterations in cognitive processes can be reflected as changes in saccadic eye movement, and it has been proposed that the analysis of certain parameters of saccadic eye movement would be useful for early detection of cognitive and motor alterations in different pathologies, including multiple sclerosis, Parkinson’s and Alzheimer’s diseases^20–23^, and also MHE^24,25^.

Some studies reported alterations in eye movement in patients with liver cirrhosis^24–26^. Alterations in eye movements such as “small pursuit eye movements” have been described in patients with HE^24^. However, although MHE patients showed a qualitative increase in corrective catch-up saccades, they did not show significant differences compared to patients with no cognitive impairment^24^. The latencies of saccades are longer in cirrhotic patients than in control subjects^25,26^, these alterations are reduced after liver transplantation^26^, and correlate with the evolution of the results obtained by these patients in psychometric tests^25,26^. These studies suggest that deeper research on ocular movements could improve the understanding of MHE alterations, and be useful for MHE diagnosis.

In this study, we performed a detailed characterization of the alterations suffered by MHE patients through the extensive analysis of 177 saccadic eye movement parameters, measured using an OSCANN desk100 gaze tracker^27^, based on video electro-oculography (VOG) techniques. It is a sensitive device that uses non-invasive technology for the fine analysis of 177 parameters derived from the recording of eye movements, with much higher accuracy and precision values (lower than 0.4 and 0.03, respectively) than other commercial eye-trackers^27^. This device fulfils the legal requirements of the European Union, including the personal data protection law. All data are analyzed automatically and a report is generated for to the clinician to evaluate in real time. The OSCANN desk 100 software includes a broad set of visual tests, each one aimed at generating different oculomotor responses, and can provide precise analysis and objective evaluations of eye movement alterations involved in several neurological diseases and their progression. Besides, the premises of these visual tests are simple enough for patients to understand and perform them easily.

The main aim of this work was to characterize the alterations in saccadic eye movements in patients with MHE compared with healthy controls and cirrhotic patients without MHE (NMHE). We also assessed the relationship of eye movements impairment with some neurological alterations, and the utility of eye movements as a diagnostic tool for MHE.

We analyzed 177 parameters of eye movements using a set of visual tests (visually guided saccades, memory guided saccades, anti-saccades, smooth pursuit eye movements and fixation test) measured by the OSCANN desk 100 device. We assessed their diagnostic capacity for MHE, and correlated with cognitive alterations associated to MHE.

## Results

### Neuropsychological performance

The PHES battery classified 32 patients (27%) as “with MHE” and 86 (73%) patients as “without MHE” (NMHE). The study also included 35 healthy control subjects without liver disease. MHE patients showed reduced performance in all subtests from PHES and in the three tasks from Stroop test, compared to NMHE patients and controls (Table 1). No significant differences were observed in age or in severity of liver damage (Child Pugh and MELD scores; Table 1).

**Table 1:** Study sample data and performance of controls and patients in the neuropsychological tests performed.

	Controls	NMHE patients *P* versus control	MHE patients *P* versus control	MHE patients *P* versus NMHE	Global FDR values
Number of subjects (Male/Female)	35 (23/12)	86 (71/15)	32 (23/9)		
Age^c^	62 ± 1.7	62 ± 0.94	65 ± 1.5	ns	ns
**Etiology**					
Alcohol		37	13		
VHC/VHB		20/1	5/1		
VHC + alcohol/VHB + alcohol		4/1	2/0		
NASH		12	6		
MAFLD		7	2		
Cryptogenic		2	2		
Autoimmune		1	0		
Others		1	1		
Child Pugh score (A/B/C)		70/16/0	24/8/0	ns	
MELD score		9.2 ± 0.4	9.4 ± 0.570	ns	
**PHES corrected scores**					
PHES global score^c^	0.6 ± 0.31	− 0.5 ± 0.16**	− 6.9 ± 0.55***	< 0.001	< 0.001
DST^c^	0.1 ± 0.07	0.05 ± 0.04	− 0.66 ± 0.15***	< 0.001	< 0.001
NCT-A^c^	0.3 ± 0.09	0.16 ± 0.05	− 1.1 ± 0.21***	< 0.001	< 0.001
NCT-B^c^	0.4 ± 0.13	0.12 ± 0.05*	− 1.3 ± 0.19***	< 0.001	< 0.001
SD^c^	0 ± 0.05	− 0.27 ± 0.05*	− 1.5 ± 0.19***	< 0.001	< 0.001
LTT^c^	− 0.2 ± 0.16	− 0.49 ± 0.1	− 2.4 ± 0.14***	< 0.001	< 0.001
**PHES raw scores**					
DST (items completed)^b^	39 ± 2.2	33 ± 1.2*	18 ± 1.2***	< 0.001	< 0.001
NCT-A (seconds)^c^	32 ± 2.3	38 ± 1.6*	101 ± 18***	< 0.001	< 0.001
NCT-B (seconds)^c^	82 ± 8.6	105 ± 4.6**	228 ± 21***	< 0.001	< 0.001
SD (seconds)^c^	60 ± 2.5	73 ± 2.4**	112 ± 8.2***	< 0.001	< 0.001
LTT (seconds + errors)^c^	108 ± 6	123 ± 4.2	212 ± 13***	< 0.001	< 0.001
**Stroop test**					
Congruent task (words)^a^	109 ± 3	100 ± 2.1	78 ± 2.7***	< 0.001	< 0.001
Neutral task (colors)^a^	83 ± 2.4	74 ± 1.4**	57 ± 1.6***	< 0.001	< 0.001
Incongruent task (items)^a^	46 ± 2	42 ± 1.1	30 ± 1.4***	< 0.001	< 0.001

Values are expressed as mean ± SEM. Abbreviations: NMHE and MHE, patients without and with minimal hepatic encephalopathy according to PHES. MELD (Model for End-Stage Liver Disease) and Child Pugh Scores measure the severity of chronic liver disease. The higher the score is, the more severe the liver disease. VHB, VHC, hepatitis B and C viruses, respectively; NASH, Non-alcoholic steatohepatitis; MAFLD, Metabolic-associated fatty liver disease; PHES, Psychometric Hepatic Encephalopathy Score; DST, Digit Symbol Test; NCT-A, NCT-B: Number Connection Test A and B; SD, Serial Dotting Test; LTT, Line Tracing Test. Differences between groups were analyzed using one of three possibilities: one-way ANOVA followed by Tukey’s multiple comparison test (a) parametric and homoscedastic variables, Welch’s ANOVA followed by Games-Howell’s multiple comparison test (b) for parametric but non-homoscedastic variables, and Kruskal–Wallis followed by Dunn’s multiple comparison test (c) for non-parametric variables. Differences in MELD and Child Pugh scores were analyzed by unpaired t-test and Fisher's exact test, respectively. Significant differences compared to controls are indicated by asterisks: *p < 0.05; **p < 0.01; ***p < 0.001; ns, non-significant.

### Analysis of 177 parameters of 10 eye movements tests

Different types of eye movements were analysed: visually-guided saccades, memory-guided saccades, anti-saccades and smooth pursuit, and fixation test. For each of these tests a number of parameters were analysed: latency, velocity, positive and negative error, etc.; resulting in the exhaustive analysis of 177 parameters of 10 different eye movements tests. The values for 165 of these parameters are shown in Tables 2, 3, and 4 and Tables S2 and S3. The lacking parameters are reiterative.

**Table 2:** Results of eye movement tests in the three groups of study. Horizontal and vertical antisaccades.

	Controls	NMHE patients *P* versus control	MHE patients *P* versus control	MHE patients *P* versus NMHE	Global FDR values
**Horizontal antisaccades**					
Latency (ms)^c^	360 ± 10	439 ± 20*	590 ± 68**	0.040	0.029
Standard deviation of latency^c^	83 ± 11	99 ± 10	68 ± 14	ns	ns
Latency of reflexive saccades (ms)^c^	560 ± 24	652 ± 25*	809 ± 50***	0.004	0.003
SD of latency of reflexive saccades^c^	156 ± 22	173 ± 14	203 ± 20*	ns	ns
Duration of reflexive saccades (ms)^c^	216 ± 15	283 ± 18*	375 ± 37***	0.019	0.009
SD of duration of reflexive saccades^c^	101 ± 8.3	151 ± 13*	212 ± 27**	0.018	0.009
Positive error^c^	2.9 ± 0.47	3.9 ± 0.4	5.5 ± 0.67**	0.018	0.009
Standard deviation of positive error^c^	1.7 ± 0.35	2.4 ± 0.24*	3.7 ± 0.41***	0.010	0.003
Negative error^c^	− 2.8 ± 0.42	− 4.5 ± 0.51*	− 6.1 ± 0.96**	0.027	0.009
Standard deviation of negative error^c^	2 ± 0.31	3.1 ± 0.38	6 ± 1.1***	0.006	0.009
Blinks^c^	5.5 ± 0.81	4.1 ± 0.61	6.2 ± 1.2	ns	ns
Number of correct antisaccades^c^	3.9 ± 0.6	2.6 ± 0.32	1.2 ± 0.38***	0.005	0.009
Number of corrected antisaccades^c^	5.6 ± 0.65	5.6 ± 0.4	4.5 ± 0.64	ns	ns
Number of incorrect antisaccades^c^	1.2 ± 0.42	1.8 ± 0.35	3.7 ± 0.75**	0.003	0.009
Number of second order reflexive saccades^c^	0.11 ± 0.05	0.14 ± 0.06	0.3 ± 0.09*	0.019	ns
Number of anticipated antisaccades^c^	0.97 ± 0.32	1.4 ± 0.23	1.7 ± 0.34	ns	ns
Return latency (ms)^c^	334 ± 20	395 ± 14*	376 ± 20	ns	ns
SD of return latency^c^	100 ± 6.7	133 ± 8.6	153 ± 18	ns	ns
Return peak of velocity (°/ms)^c^	324 ± 20	347 ± 13	320 ± 17	ns	ns
SD of return peak of velocity^c^	174 ± 11	172 ± 7.5	165 ± 9.1	ns	ns
**Vertical antisaccades**					
Latency (ms)^b^	381 ± 13	428 ± 14	597 ± 50***	< 0.001	0.004
Standard deviation of latency^c^	81 ± 11	97 ± 10	132 ± 29	ns	ns
Latency of reflexive saccades (ms)^c^	549 ± 23	577 ± 20	924 ± 78***	< 0.001	< 0.001
SD of latency of reflexive saccades^c^	93 ± 9.6	122 ± 12	156 ± 26	ns	ns
Duration of reflexive saccades (ms)^c^	184 ± 11	234 ± 16	301 ± 40*	ns	ns
SD of duration of reflexive saccades^c^	78 ± 9.2	118 ± 9.9*	105 ± 13	ns	ns
Positive error^c^	1.5 ± 0.18	2.2 ± 0.2	3.1 ± 0.33***	0.009	0.014
SD of positive error^c^	0.96 ± 0.14	1.3 ± 0.14	2 ± 0.24**	0.021	0.041
Negative error^c^	− 2.4 ± 0.27	− 3.4 ± 0.31	− 5.6 ± 0.82**	0.009	0.016
SD of negative error^c^	1.6 ± 0.19	2.1 ± 0.27	4.2 ± 0.71*	0.016	ns
Blinks^c^	4.1 ± 0.71	3.2 ± 0.36	4.4 ± 0.74	ns	ns
Number of correct antisaccades^c^	2.4 ± 0.36	1.8 ± 0.21	1.1 ± 0.27*	ns	ns
Number of corrected antisaccades^c^	3.8 ± 0.4	3.7 ± 0.28	2.5 ± 0.41*	0.035	ns
Number of incorrect antisaccades^c^	0.74 ± 0.29	1.1 ± 0.23	2.3 ± 0.46***	< 0.001	0.004
Number of second order reflexive saccades^c^	0.2 ± 0.08	0.18 ± 0.06	0.31 ± 0.1	ns	ns
Number of anticipated antisaccades^c^	0.63 ± 0.22	0.87 ± 0.15	1.3 ± 0.23**	0.019	0.041
Return latency (ms)^c^	349 ± 16	382 ± 12	377 ± 24	ns	ns
SD of return latency^c^	84 ± 8.8	112 ± 8.2	119 ± 15	ns	ns
Return peak of velocity (°/ms)^c^	222 ± 13	209 ± 9.8	221 ± 17	ns	ns
SD of return peak of velocity^c^	119 ± 8.2	102 ± 4.7	143 ± 12	0.002	0.019

All values are expressed as mean ± SEM. Abbreviations: NMHE and MHE, patients without and with minimal hepatic encephalopathy according to PHES; SD, Standard deviation. Differences between groups were analyzed using one of three possibilities: one-way ANOVA followed by Tukey’s multiple comparison test (a) parametric and homoscedastic variables, Welch’s ANOVA followed by Games-Howell’s multiple comparison test (b) for parametric but non-homoscedastic variables, and Kruskal–Wallis followed by Dunn’s multiple comparison test (c) for non-parametric variables. Significant differences compared to controls are indicated by asterisks: *p < 0.05; **p < 0.01; ***p < 0.001; ns, non-significant. Detailed definitions of eye movement variables are in Supporting Information.

**Table 3:** Results of eye movement tests in the three groups of study. Horizontal, vertical, and sinusoidal smooth pursuit.

	Controls	NMHE patients *P* versus control	MHE patients *P* versus control	MHE patients *P* versus NMHE	Global FDR values
**Horizontal smooth pursuit**					
Blinks^c^	2.9 ± 0.58	2.4 ± 0.39	3.7 ± 0.62	0.025	ns
Catch-up saccades^b^	22 ± 1.7	22 ± 0.79	24 ± 1.8	ns	ns
Back-up saccades^c^	0.39 ± 0.14	0.8 ± 0.2	1.3 ± 0.29**	0.004	0.025
Square wave jerks^c^	3.3 ± 0.52	4.8 ± 0.52	5.1 ± 0.55*	ns	ns
Pursuit time (%)^c^	92 ± 0.72	90 ± 0.66	89 ± 0.65*	0.030	ns
Latency (ms)^c^	307 ± 13	328 ± 15	311 ± 22	ns	ns
Total mean squared error of position^c^	2.3 ± 0.24	3.2 ± 0.26	3.8 ± 0.36**	0.043	0.037
Pursuit mean squared error of position^c^	2.3 ± 0.23	3.1 ± 0.26	3.7 ± 0.35**	0.044	0.037
Gain^c^	0.86 ± 0.02	0.81 ± 0.02	0.74 ± 0.03**	0.008	0.025
Pursuit mean squared error of velocity^c^	11 ± 0.87	9.4 ± 0.28	11 ± 1	ns	ns
**Vertical smooth pursuit**					
Blinks^c^	3.5 ± 0.7	2.6 ± 0.38	4.9 ± 0.87	0.045	ns
Catch-up saccades^b^	14 ± 1.2	14 ± 0.69	18 ± 0.81*	0.009	0.006
Back-up saccades^c^	0.77 ± 0.27	1.2 ± 0.21	1.8 ± 0.4	ns	ns
Square wave jerks^c^	1.9 ± 0.46	3 ± 0.35*	3.1 ± 0.4*	ns	ns
Pursuit time (%)^c^	92 ± 0.9	92 ± 0.65	89 ± 0.98*	0.023	ns
Latency (ms)^c^	376 ± 33	357 ± 15	402 ± 33	ns	ns
Total mean squared error of position^c^	1.8 ± 0.22	2.3 ± 0.21	2.7 ± 0.23**	0.011	0.021
Pursuit mean squared error of position^c^	1.6 ± 0.16	2 ± 0.15	2.5 ± 0.18**	0.004	0.012
Gain^a^	0.84 ± 0.03	0.8 ± 0.02	0.68 ± 0.03***	0.003	0.005
Pursuit mean squared error of velocity^c^	7.4 ± 0.6	7.7 ± 0.48	8.1 ± 0.58	ns	ns
**Sinusoidal smooth pursuit**					
Blinks^c^	1.7 ± 0.45	2.9 ± 0.42	3 ± 0.57	ns	ns
Catch-up saccades^a^	18 ± 1.6	17 ± 1	21 ± 1.4	0.023	ns
Back-up saccades^c^	0.57 ± 0.19	0.68 ± 0.12	1.2 ± 0.34	ns	ns
Square wave jerks^c^	3.1 ± 0.74	4.1 ± 0.52	4.7 ± 0.78	ns	ns
Pursuit time (%)^c^	93 ± 0.8	92 ± 0.64	90 ± 0.8*	ns	ns
Latency (ms)^c^	257 ± 9.9	276 ± 7.4	313 ± 11***	0.001	0.005
Total mean squared error of position^c^	2.4 ± 0.36	3 ± 0.24	4 ± 0.42**	0.013	0.022
Pursuit mean squared error of position^c^	2.4 ± 0.34	2.8 ± 0.23	3.9 ± 0.41**	0.013	0.022
Gain^b^	0.89 ± 0.03	0.8 ± 0.03	0.67 ± 0.05**	ns	0.005
Pursuit mean squared error of velocity^c^	9 ± 0.38	9.5 ± 0.38	10 ± 0.59	ns	ns

All values are expressed as mean ± SEM. Abbreviations: NMHE and MHE, patients without and with minimal hepatic encephalopathy according to PHES. Differences between groups were analyzed using one of three possibilities: one-way ANOVA followed by Tukey’s multiple comparison test (a) parametric and homoscedastic variables, Welch’s ANOVA followed by Games-Howell’s multiple comparison test (b) for parametric but non-homoscedastic variables, and Kruskal–Wallis followed by Dunn’s multiple comparison test (c) for non-parametric variables. Significant differences compared to controls are indicated by asterisks: *p < 0.05; **p < 0.01; ***p < 0.001; ns, non-significant. Detailed definitions of eye movement variables are in Supporting Information.

**Table 4:** Results of eye movement tests in the three groups of study.

	Controls	NMHE patients *P* versus control	MHE patients *P* versus control	MHE patients *P* versus NMHE	Global FDR values
**Fixation**					
Blinks^c^	3.9 ± 0.74	3.5 ± 0.48	5.2 ± 0.83	0.024	ns
Saccades^c^	1.5 ± 0.35	3.8 ± 0.62*	5.5 ± 1.4**	ns	ns
Microsaccades^c^	6.5 ± 0.99	8.3 ± 1	11 ± 2.1	ns	ns
Number of drifts^c^	1.7 ± 0.46	2.8 ± 0.6	4.9 ± 1.2*	0.038	ns
Monophasic- square wave jerks^c^	1.8 ± 0.52	2.1 ± 0.38	2.7 ± 0.57	ns	ns
Biphasic- square wave jerks^c^	0.61 ± 0.29	1.2 ± 0.31	2.1 ± 0.56*	ns	ns
Distractions^c^	0.57 ± 0.28	0.3 ± 0.09	0.77 ± 0.32	ns	ns
Bcea (°)^c^	0.65 ± 0.12	1.5 ± 0.29	1.6 ± 0.26**	0.004	ns
Ox (horizontal standard deviation) (°)^c^	0.29 ± 0.02	0.59 ± 0.09	0.7 ± 0.13**	0.017	ns
Oy (vertical standard deviation) (°)^c^	0.33 ± 0.05	0.45 ± 0.05	0.51 ± 0.06**	0.018	ns
Centroid x (horizontal) (°)^c^	− 0.07 ± 0.05	− 0.06 ± 0.06	− 0.04 ± 0.03	ns	ns
Centroid y (vertical) (°)^b^	0.06 ± 0.08	0.06 ± 0.09	− 0.26 ± 0.18	ns	ns
Microsaccades amplitude (°)^c^	0.32 ± 0.02	0.4 ± 0.02*	0.44 ± 0.02***	0.030	0.023
SD of microsaccades amplitude^c^	0.12 ± 0.01	0.17 ± 0.02*	0.19 ± 0.02*	ns	ns
Velocity of microsaccades (°/s)^c^	13 ± 0.8	14 ± 0.52	16 ± 0.41**	0.009	ns
SD of velocity of microsaccades^c^	2.5 ± 0.31	3.5 ± 0.32	3.4 ± 0.37	ns	ns
Peak velocity-microsaccades (°/s)^b^	22 ± 1.3	25 ± 1.1	28 ± 0.83**	ns	0.008
SD of peak velocity-microsaccades^c^	5.4 ± 0.74	7.7 ± 0.79	8.1 ± 0.96*	ns	ns
Microsaccades frequency^c^	0.56 ± 0.1	0.58 ± 0.06	0.78 ± 0.1	0.036	ns
Drift amplitude (°)^c^	0.24 ± 0.03	0.21 ± 0.02	0.28 ± 0.02	0.018	ns
SD of drift amplitude^b^	0.18 ± 0.04	0.12 ± 0.01	0.17 ± 0.02	ns	ns
Drift velocity (°/s)^c^	3 ± 0.48	2.7 ± 0.22	3.4 ± 0.34	ns	ns
SD	1.2 ± 0.3	1 ± 0.16	0.86 ± 0.15	ns	ns
Peak of velocity-drift (°/s)^c^	10 ± 1.2	8.4 ± 0.7	11 ± 0.86	0.020	ns
SD of peak of velocity-drift^a^	2.8 ± 0.63	3.2 ± 0.38	3.8 ± 0.48	ns	ns
Square wave jerks—amplitude (°)^c^	0.68 ± 0.07	0.88 ± 0.08	0.81 ± 0.08	ns	ns
SD of square wave jerks—amplitude^c^	0.41 ± 0.09	0.48 ± 0.05	0.45 ± 0.06	ns	ns
Square wave jerks—time (ms)^c^	245 ± 26	274 ± 23	302 ± 24	ns	ns
SD of square wave jerks—time^c^	176 ± 19	142 ± 17	180 ± 18	ns	ns

Fixation test. All values are expressed as mean ± SEM. Abbreviations: NMHE and MHE, patients without and with minimal hepatic encephalopathy according to PHES; SD, Standard deviation. Differences between groups were analyzed using one of three possibilities: one-way ANOVA followed by Tukey’s multiple comparison test (a) parametric and homoscedastic variables, Welch’s ANOVA followed by Games-Howell’s multiple comparison test (b) for parametric but non-homoscedastic variables, and Kruskal–Wallis followed by Dunn’s multiple comparison test (c) for non-parametric variables. Significant differences compared to controls are indicated by asterisks: *p < 0.05; **p < 0.01; ***p < 0.001; ns, non-significant. Detailed definitions of eye movement variables are in Supporting Information.

Seventy-four parameters were significantly different in MHE patients compared to controls and 56 significantly different from NMHE patients (Tables 2, 3, 4).

### Visual guided saccades

**Figure 1:**
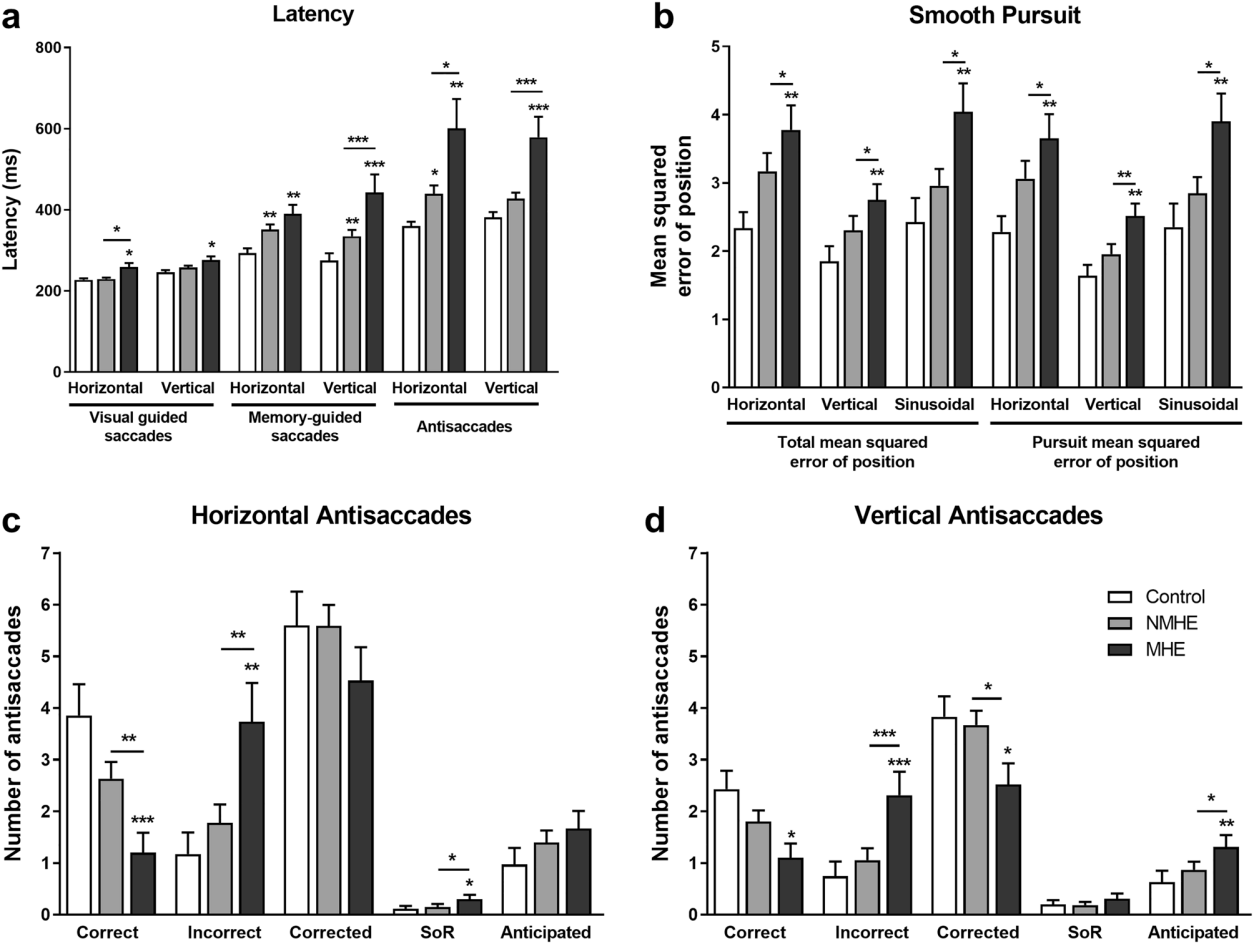
MHE patients show longer latencies and worse performance in eye movement tests. (**a**) Latencies in visual guided saccades, memory-guided saccades and antisaccades tests. (**b**) Total mean squared and pursuit mean squared error of position in the three versions of Smooth pursuit test. (**c**,**d**) Performance in horizontal and vertical antisaccades tests. MHE, NMHE: patients with and without minimal hepatic encephalopathy, respectively. SoR, second order reflexive saccades Results are the mean ± SEM. *p < 0.05; **p < 0.01; ***p < 0.001.

In visually-guided saccades tests only 3 of 32 parameters (Table S2) showed alterations allowing the differentiation of MHE patients from both NMHE patients and controls, the latency (*p* < 0.05) (Fig. 1a) and SD of latency of the horizontal version (*p* = 0.001), and the return latency of the vertical version (*p* = 0.007).

### Memory-guided saccades

For memory-guided saccades tests only 3 out of 36 parameters analyzed show alterations (Table S3) that can differentiate MHE patients from NMHE patients and controls, the gain (*p* = 0.04) and number and rate of correctly performed saccades from the horizontal version of (*p* < 0.001). It must be noted that the number (and rate) of correctly performed saccades from the vertical version of this test shows similar alterations, but in this case NMHE patients show also mild alterations. In the vertical memory-guided saccades test, MHE patients showed longer latency than NMHE patients and controls (*p* < 0.001) (Fig. 1a, and Table S3).

### Anti-saccades

For anti-saccadic movements, MHE patients show alterations in 20 of 50 parameters when compared to NMHE patients and controls (Table 2). These alterations include positive error, the standard deviation (SD) of negative error and the number of incorrect anti-saccades for both horizontal and vertical tests. In the horizontal anti-saccades test there are alterations in the number of correct anti-saccades and second order reflexive saccades (Fig. 1c, and Table 2). In the vertical anti-saccades test, the latency of anti-saccades and reflexive saccades, negative error, SD of positive error, and the number of corrected and anticipated anti-saccades are altered (Fig. 1a,d, and Table 2). As the cognitive demand of the test increases, patients with MHE show much higher latencies than the other study groups (Fig. 1a). Most of the variables altered in the vertical anti-saccades test are also altered in the horizontal anti-saccades test, but NMHE patients also show mild alterations in this case.

### Smooth pursuit

MHE patients show alterations in 13 out of 30 parameters obtained from the smooth pursuit tests when compared to NMHE patients and controls (Table 3). In the three versions of this test, the total and pursuit mean squared error of position is altered in MHE (Fig. 1b); while the catch-up saccades, pursuit time and gain are altered only in two of the three tests (Table 3). Back-up saccades are altered in the horizontal version, and latency is altered only in the version that includes velocity changes in the stimulus movement.

### Fixation

In the fixation test, 5 out of 29 analyzed parameters are altered in MHE patients compared to NMHE patients and controls (Table 4). These are the number of drifts, BCEA and both its horizontal and vertical SD, amplitude and velocity of microsaccades. In this test NMHE patients showed alterations when compared to controls, in number of saccades, and microsaccades amplitude, with a much lower level of significance than for MHE group.

### Correlations between eye movements and neuropsychological tests

We performed a correlation analysis between the eye movement parameters altered in the patients and their performance in the neuropsychological test (Table 5 and Tables S4–S8).

**Table 5:** Correlations between neuropsychological tests and antisaccades test parameters.

PHES subtests	Latency	Latency reflexive saccades	Duration reflexive saccades	Positive error	Negative error	Correct saccades	Corrected saccades	Incorrect saccades	Second order reflexive saccades	Anticipated saccades
**Horizontal antisaccades**										
PHES score										
r	− 0.232	− **0.375**	− **0.312**	− **0.352**	**0.303**	**0.468**	0.134	− **0.409**	− 0.236	− 0.137
*p*	0.034	**< 0.001**	**< 0.001**	**< 0.001**	**0.001**	**< 0.001**	ns	**< 0.001**	0.005	ns
DST (items completed)										
r	− 0.321	**− 0.474**	**0.420**	− 0.389	**0.443**	**0.503**	0.178	**− 0.435**	− 0.277	− 0.281
*p*	0.005	**< 0.001**	**< 0.001**	< 0.001	**< 0.001**	**< 0.001**	ns	**< 0.001**	0.002	0.001
NCT-A (seconds)										
r	0.337	**0.502**	**0.510**	0.333	**− 0.426**	**− 0.495**	− 0.214	**0.476**	0.248	0.213
*p*	0.003	**< 0.001**	**< 0.001**	< 0.001	**< 0.001**	**< 0.001**	0.017	**< 0.001**	0.005	0.018
NCT-B (seconds)										
r	0.358	**0.581**	0.280	0.365	**− 0.490**	**− 0.583**	− 0.125	**0.502**	0.301	0.267
*p*	0.001	**< 0.001**	0.003	< 0.001	**< 0.001**	**< 0.001**	ns	**< 0.001**	< 0.001	0.003
SD (seconds)										
r	0.196	0.346	0.350	0.287	− 0.276	− 0.396	0.083	0.221	0.291	0.028
*p*	ns	< 0.001	< 0.001	0.003	0.005	< 0.001	ns	0.014	0.001	ns
LTT (seconds + errors)										
r	0.271	0.375	**− 0.437**	0.344	− 0.380	**− 0.505**	− 0.099	**0.437**	0.199	0.162
*P*	0.020	< 0.001	**< 0.001**	< 0.001	< 0.001	**< 0.001**	ns	**< 0.001**	0.027	ns
Stroop-incongruent task										
r	− 0.278	**− 0.377**	**− 0.324**	− 0.242	**0.343**	**0.542**	0.09	**− 0.376**	− 0.199	− 0.239
*p*	0.01	**< 0.001**	**< 0.001**	0.01	**< 0.001**	**< 0.001**	ns	**< 0.001**	0.02	0.006
**Vertical antisaccades**										
PHES score										
r	**− 0.357**	**− 0.414**	− 0.266	**− 0.405**	− 0.305	**0.436**	0.1620	**− 0.410**	− 0.151	− 0.220
*p*	**< 0.001**	**< 0.001**	0.005	**< 0.001**	0.011	**< 0.001**	ns	**< 0.001**	ns	0.010
DST (items completed)										
r	− 0.356	**− 0.560**	− 0.155	**− 0.456**	− 0.284	**0.453**	0.262	**− 0.489**	− 0.067	− 0.314
*p*	0.002	**< 0.001**	ns	**< 0.001**	0.029	**< 0.001**	0.003	**< 0.001**	ns	< 0.001
NCT-A (seconds)										
r	**0.419**	**0.536**	0.234	0.331	0.235	**− 0.454**	− 0.221	**0.491**	0.065	0.238
*p*	**< 0.001**	**< 0.001**	0.029	0.002	ns	**< 0.001**	0.014	**< 0.001**	ns	0.008
NCT-B (seconds)										
R	**0.417**	**0.581**	0.271	0.383	0.203	**− 0.509**	− 0.178	**0.414**	0.146	0.408
*p*	**< 0.001**	**< 0.001**	0.013	< 0.001	ns	**< 0.001**	ns	**< 0.001**	ns	< 0.001
SD (seconds)										
r	0.397	0.348	0.033	0.302	0.275	− 0.361	− 0.113	0.323	0.158	0.111
*p*	< 0.001	< 0.001	ns	0.006	0.035	< 0.001	ns	< 0.001	ns	ns
LTT (seconds + errors)										
r	0.376	**0.404**	0.081	**0.419**	0.267	**− 0.448**	− 0.199	**0.408**	0.162	0.229
*p*	0.001	**< 0.001**	ns	**< 0.001**	0.040	**< 0.001**	0.028	**< 0.001**	ns	0.011
Stroop-incongruent task										
r	− 0.314	**− 0.486**	− 0.300	− 0.310	**0.333**	**0.419**	0.168	**− 0.418**	− 0.158	− 0.266
*p*	0.005	**< 0.001**	0.002	0.003	**< 0.001**	**< 0.001**	ns	**< 0.001**	ns	0.002

Spearman correlation parameters are shown. PHES, Psychometric Hepatic Encephalopathy Score; DST, Digit Symbol Test; NCT-A, NCT-B: Number Connection Test A and B; SD, Serial Dotting Test; LTT, Line Tracing Test; ns, not significant. More significant correlations are highlighted in bold.

**Figure 2:**
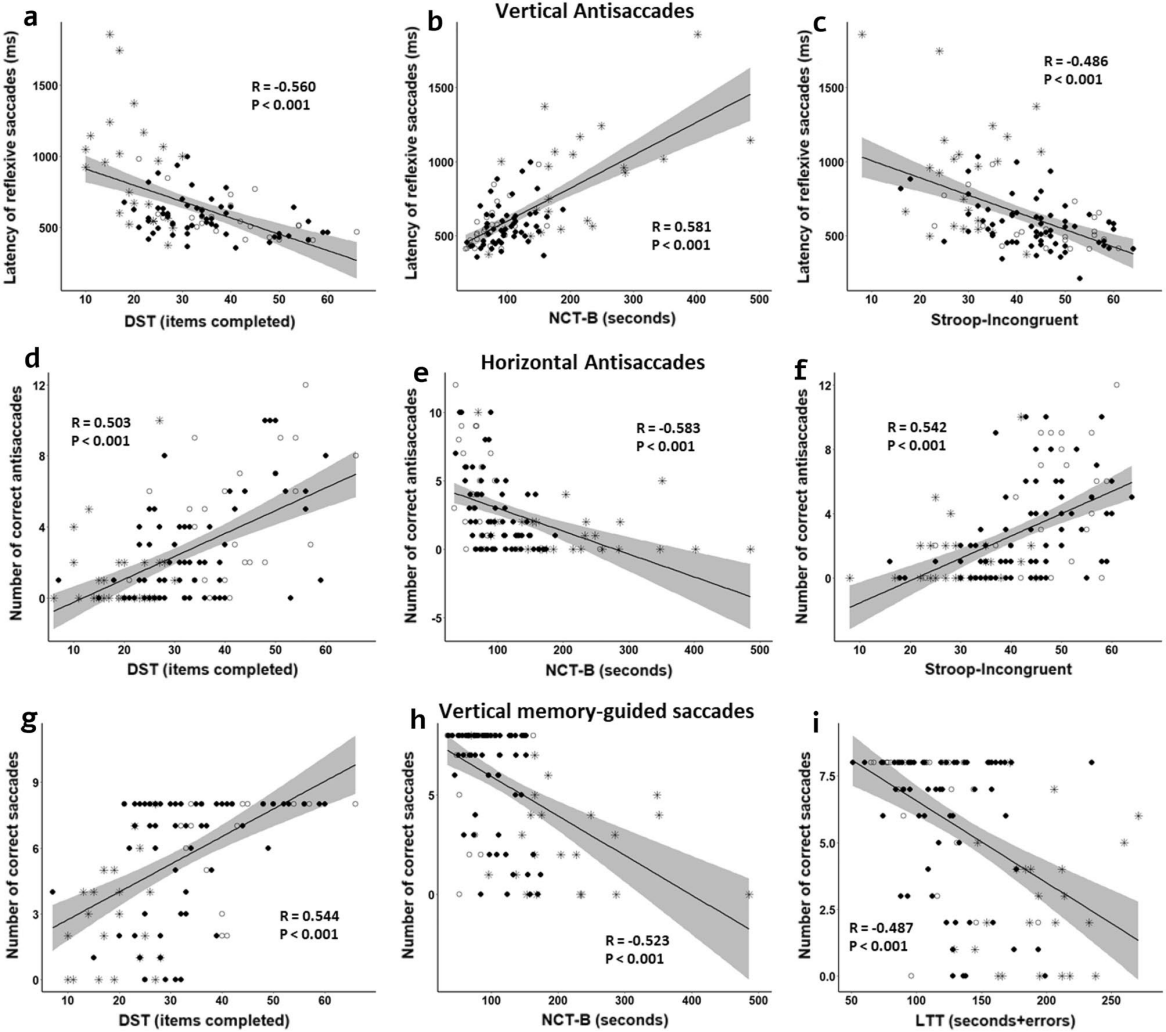
Correlations between eye movement tests and cognitive performance. (**a**–**c**) Correlations of Latency of reflexive saccades in vertical antisaccades test with subtests from PHES and Stroop test. (**d**–**f**) Correlations of correct antisaccades in horizontal antisaccades test with subtests from PHES and Stroop test. (**g**–**i**) Correlations of correct saccades in vertical memory-guided saccades test with subtests from PHES. Spearman correlation parameters (R and P) are shown. DST, Digit Symbol Test; NCT-B: Number Connection Test B; SD, LTT, Line Tracing Test. Each study group is indicated with different symbols: white circles (controls), black circles (NMHE patients) and stars (MHE patients).

There were significant correlations between PHES score and subtests from PHES with parameters of anti-saccades test (Table 5). In general, for both versions of this test, the best correlations were found with PHES subtests measuring mental processing speed (DST) and attention (NCT-A and NCT-B) and also with visuo-spatial tracing performance (LTT) (Fig. 2a,b,d,e, and Table 5). An altered performance in antisaccades test also correlated with poorer performance in Stroop test (Fig. 2c,f, and Table 5).

There were significant correlations between PHES score and most of parameters of fixation test, highlighting the amplitude and velocity of microsaccades, which also correlate with subtests from PHES (Table S4). The best correlations in fixation test were found with subtests measuring mental processing speed (DST) and attention (NCT-A and NCT-B).

Correct saccades and latency in both horizontal and vertical memory-guided tests showed significant correlations with PHES score, and also with DST, NCT-A, NCT-B, and LTT subtests (Fig. 2g–i and Table S5).

Regarding smooth pursuit tests, there were significant correlations of PHES score with total and pursuit mean squared error of position and gain in both modalities of this test (Table S6), and also in sinusoidal smooth pursuit test (Table S7), and with back-up saccades in horizontal smooth pursuit test (Table S6). These parameters also correlated significantly with most PHES subtests.

Finally, in the visually-guided saccades test, there were better correlations with PHES and its subtests in the vertical version of test, mainly in Latency and return latency parameters. SD of latency is the parameter with the best correlations in the horizontal version of this test (Table S8).

### Diagnostic capacity of visual tests for MHE

To assess if some parameters from visual tests could serve to diagnose MHE, we performed Receiver operating characteristic (ROC) curves with PHES score as the reference.

In vertical antisaccades test, Latency of reflexive saccades was the best parameter discriminating MHE and NMHE patients, with an AUC (area under ROC curve) of 0.776 (*p* < 0.0001), followed by the latency (AUC: 0.718; *p* = 0.01) and the negative error (AUC: 0.698; *p* = 0.004).

In the memory-guided saccades test, the correct saccades showed an AUC of 0.747 (*p* = 0.0001) in the horizontal version, and an AUC of 0.789 (*p* < 0.0001) in the vertical version of the test. Latency in vertical version also showed a significant AUC: 0.715 (*p* = 0.01).

## Discussion

In this study, we performed an exhaustive characterization of eye movements by video electro-oculography (VOG) in patients with liver cirrhosis with or without MHE, and controls. MHE patients showed alterations in the visual tests performed compared to patients without MHE and controls, and most altered parameters correlate with alterations in attention and mental processing speed, measured by psychometric tests.

In recent years, VOG techniques have been gaining relevance as methods for early detection of cognitive and motor dysfunctions in pathologies such as Parkinson’s or Alzheimer’s disease, or multiple sclerosis^20–23^. These techniques have already been used to study of both minimal and overt hepatic encephalopathy, and alterations similar to those observed in other pathologies have been reported^24–26^. The current study provides substantial advances in this matter. The development and application of a test battery that can analyse 177 variables from 10 different kinds of eye movements tests in no more than 20 min provides a new level of detail to the detection of the aforementioned alterations, as well as new ways in which VOG can be applied in the clinical research field.

When observed globally, MHE patients show significant alterations that can differentiate them from both healthy individuals and NMHE patients in 56 of the 177 total variables analysed. Only 18 variables can differentiate controls from patients with liver cirrhosis, indicating a clear association of the remaining alterations with neurological impairment.

As the cognitive demand of the test increases, more parameters are altered in MHE and NMHE.

Parameters significantly altered in MHE patients when compared to both NMHE patients and controls are fairly distributed across most tests. Latency is usually affected in most tests. With the exception of the horizontal and vertical smooth pursuit tests, MHE patients have increased latencies when compared to NMHE patients who have increased latencies when compared to controls. This impairment is especially patent in vertical anti-saccades, memory-guided saccades, horizontal visually-guided saccades and sinusoidal smooth pursuit. These results agree with Cunniffe et al.^25^, who found prolonged saccadic latencies and its variability in cirrhotic patients with covert hepatic encephalopathy (CHE) compared to non-CHE patients, using a visual-guided saccades test in a horizontal version. We also found that latency and its standard deviation were higher in MHE than in NMHE patients, in the visually-guided test. Latency alterations found in MHE suggest impaired processing of visual stimuli and response to them, which causes the delayed response. MHE patients show impaired mental processing speed and attention, which correlate with longer latencies in visual tests.

Other parameters analysed in several tests are those measuring eye movements precision (Positive and Negative error). Significant alterations are consistently found in the anti-saccades tests, where MHE patients show increased errors performed and for the standard deviation of errors. This suggests that MHE patients have greater difficulties to correctly calculate and position their gaze in the opposite position of the presented stimulus, with either excessive or insufficient saccadic movements. The increased standard deviation suggests that such imprecise movements are less consistent during the same test. While NMHE patients or control subjects deviate a consistent distance from the desired position, MHE patients seem to perform both slightly and notably inaccurate saccadic movements. These alterations in error values would reflect impaired accuracy by MHE patients, while the increased standard deviation of such variables would indicate a lack of precision.

This lack of both accuracy and precision does not consistently appear in the memory-guided saccades tests, in which the measured eye movements are similarly directed towards a place with no present stimulus. This discrepancy would mean that, to correctly directing the gaze towards a place with no present stimulus, the recent memory of the presence of such stimulus is sufficient. The task of correctly calculating the mirrored position of a visual stimulus while averting the gaze from the stimulus itself, with no other frame of reference, in the antisaccades task, is quite different and, as the present results suggest, some of the processes involved are impaired in MHE patients. Processes such as inhibitory control and working memory are involved in successful responses in antisaccades test^27^. These cognitive functions are impaired in patients with MHE^7,28–30^. The active suppression of the predominant saccade towards the target, and the active fixation on the spatial location of the end point of the antisaccade depend mainly on the inhibition system. MHE patients show impaired inhibitory control^7,28,29^, which could in part account for the alterations found in antisaccades task, given the correlations found between a poor performance in the Stroop test and altered parameters of antisaccades test.

A poor performance in both the anti-saccades tests and the memory-guided saccades tests in MHE patients could be related to impaired mental processing speed and attention, given the correlations with psychometric tests assessing these functions. These impairments would account for the longer latencies observed and for the decreased number of correctly performed eye movements in MHE patients.

MHE patients show a reduced functional connectivity in attention-related networks such as the Default Mode Network, the salience network, and Left Frontoparietal Network^31^. These brain areas are responsible for attention working memory, and executive control, all necessary for the correct execution of PHES and eye movement tests^32,33^.

Dorsolateral prefrontal cortex (DLPFC) controls the top-down decisional process determining whether to make a prosaccade by signals to the superior colliculus^22^. The functional connectivity between precuneus and DLPFC, within the cognitive control network (CCN) was reduced in patients with MHE compared to patients without MHE^34^. Moreover, we previously showed a reduction of cortical thickness in the precuneus in MHE patients compared with NMHE and control subjects^35^. Precuneus is primarily involved in visuospatial coordination, higher-order cognitive tasks, and conscious information processing. It is also selectively associated with other parietal areas involved in visuospatial information processing^36^.

Memory-related eye movements are closely related to hippocampus^37^. We previously showed structural and functional connectivity disturbances in hippocampal structures in MHE patients^38^ which could account for their poorer performance in the memory-guided tests.

The smooth pursuit tests provide insight of different processes than the other tests of the battery. In the three test versions there is a clear increase of the mean squared error of position of MHE patients. As previously discussed in other tests, this alteration indicates an overall less accurate gaze positioning in MHE patients when following the stimulus across the visual field. Moreover, the decreased gain and increased number of both catch-up and back-up saccades observed in MHE patients, together with the increased error of position, indicate an impaired capacity to consistently focus the gaze upon the stimulus position to follow its pace across the visual field. Our results agree with Montagnese et al.^24^ who found alterations in smooth pursuit eye movements in patients with MHE, but unlike these authors, in our study, by using VOG techniques, we were able to quantify and detect significant differences between patients with and without MHE.

Finally, regarding the fixation test, MHE patients show increased BCEA, together with increased standard deviation of the horizontal and vertical coordinates of their gaze focus. Although in this test the objective is to remain focused on an immobile stimulus, these alterations concur with those already discussed for other tests. An increased BCEA indicates that in MHE patients, their gaze is consistently positioned further away from the stimulus than in the other study groups, while the increased standard deviation suggests a less consistent level of error in this positioning. In this test an increase of all involuntary eye movements can be observed, although only the increased number of drifts is significant. Similarly, a significant increase in the amplitude and velocity of micro-saccades was observed. The increased amount and amplitude of involuntary eye movements would be consistent with an impaired ability to inhibit automatic movements.

Concerning the fixation test and the parameters related to the involuntary eye movements performed during it, the increased velocity that MHE patients show for all these movements, significantly higher when measuring the velocity of micro-saccades, is incongruent with the peak of velocity that, although rarely significant, this group shows in all voluntary eye movements of all other tests, in which it is either equal or slightly lower than that observed in NMHE patients and controls. Altogether, this information would suggest that the ability to rapidly transmit motor orders to the eye muscles is not impaired in MHE patients.

It must be also noted that there are parameters that can differentiate both MHE and NMHE patients from healthy individuals. This suggests that the analysis of eye movements can detect the early onset of the cognitive impairment associated with MHE at earlier stages than the PHES battery, which is the diagnostic method currently used. The separation of patients who suffer MHE from those who don’t is not a clear line. Patients may suffer mild impairment of cognitive and motor functions before they can be properly diagnosed with MHE, as supported by the slightly non-significant poorer performance of NMHE patients in many parameters and in the PHES subtests being an indication of this. This suggests that some NMHE patients may already show cognitive impairment and the analysis of eye movements appears to be able to detect this slight impairment. In this sense, we and other groups have reported that the PHES battery leaves an important number of MHE patients undiagnosed^28–30,39^.

In conclusion, the analysis and characterization of the alterations in eye movements observed in MHE patients can serve not only to further understand what cognitive processes are impaired in these patients, but also could help to develop a new, reliable and objective tool that correctly diagnoses patients who suffer from liver cirrhosis in earlier stages of their cognitive impairment to help preventing and reverting this impairment in a more easily and safely way.

## Materials and methods

### Study population

One hundred and eighteen patients with liver cirrhosis were recruited in the outpatient’s clinics of Hospital Clínico and Hospital Arnau de Vilanova, Valencia, Spain. The diagnosis of liver cirrhosis was based on clinical, biochemical, and ultrasonographic data. Exclusion criteria were overt HE or history of overt HE, recent (< 6 months) alcohol intake, infection, recent (< 6 weeks) antibiotic use or gastrointestinal bleeding, use of drugs affecting cognitive function, hepatocellular carcinoma, or neurological or psychiatric disorder. Thirty-five healthy volunteers were included in the study after discarding liver disease by clinical, analytical, and serologic analysis.

All participants were included after signing a written informed consent. Study protocols were approved by the Scientific and Ethical Committees (No. 2017/291) of Hospitals Clínico and Arnau de Vilanova, Valencia, Spain, and were in accordance with the ethical guidelines of the Helsinki Declaration.

The procedure was as follows: (1) recruitment of patients and controls, collecting written informed consent and analytical data; (2) Neuropsychological assessment and (3) eye movements evaluation. Eye movements were measured less than 1 week after neuropsychological measures.

### Neuropsychological assessment

All participants performed the Psychometric Hepatic Encephalopathy Score (PHES) battery, used for diagnosis of MHE. PHES includes five subtests: Digit Symbol test (DST), number connection test A and B (NCT-A and NCT-B), Serial Dotting test (SD), and Line Tracing test (LTT)^18,19^. The score obtained from each subtest was adjusted for age and educational level using Spanish normality tables (http://www.redeh.org/TEST_phes.htm). Patients were classified as MHE when their score was ≤  − 4 points^18^.

In order to evaluate selective attention, psychomotor speed, cognitive flexibility and inhibitory mental control, all participants performed the Stroop test, in a colour-word version, performing the congruent, neutral and incongruent tasks, as described in^7^. The number of items correctly named was adjusted for age according to Spanish normality tables.

### Analysis of eye movements

Eye movements were assessed with an OSCANN desk100 equipment from AURA Innovative Robotics (see Fig. S1). OSCANN desk100 is a novel gaze-tracker^40^ designed for clinical practice use. It is based on VOG technology, and its infrared camera captures images at 100 frames per second. The measurements are made over the dominant eye of the subject (see Supporting information for technical characteristics and software description).

A total of 10 tests were performed in the following order: visually guided saccades test, memory guided saccades test, anti-saccades test, smooth pursuit eye movements, and fixation test (Fig. S2). All tests included a horizontal and a vertical version, with the exception of the smooth pursuit test, which included an additional horizontal version in which the stimulus changes its velocity; and the fixation test. Performing the 10 tests to obtain these 177 parameters takes from 15 to 20 min per subject approximately. Recalibration of the recording camera was performed every 2 or 3 tests to ensure the accuracy of the measurement, and to allow the patient to take a brief break if needed.

The stimulus was a dot 2 cm diameter green dot over a black background. All horizontal versions are performed across the central horizontal axis of the screen, and all vertical versions are performed across the central vertical axis of the screen. The visual field is ± 20° in horizontal and ± 12° in vertical.

In the visually guided saccade tests the stimulus appears in the centre of the screen during 1500 ms and then jumps to a random location on one side of the screen, where it remains the same amount of time (see Fig. S2a). Then, the stimulus appears back in the centre of the screen, and the sequence is repeated. The subject must follow the stimulus. Each version of the test lasts 36 s. Variables measured include response latency when the stimulus moves to a side of the screen (latency), response latency when the stimulus moves back to the centre of the screen (return latency), gain (ratio between stimulus and gaze amplitude), accuracy (hypermetria, identified as positive error, or hypometria, identified as negative error), number of blinks, peak of velocity and anticipated saccades (saccades performed within 80 ms since the stimulus moved) (see Fig. S3a).

In the memory guided saccades tests, the subjects are inquired to remember the position of the stimulus (see Fig. S2b). In this case the stimulus firstly appears in the centre of the screen for 1500 ms, then moves to a random location on one side of the screen and returns back to the centre of the screen, similarly to the visually guided saccade tests. In this initial phase the subject must perform a visually guided saccade towards the places where the stimulus is located. At this point, the stimulus disappears from the screen for another 1500 ms, and the subject must perform a memory guided saccade towards the last side of the screen where the stimulus moved. Each version of this test takes 72 s. The variables analyzed include the same as in the visually guided saccades, as well as the number of correct memory saccades performed.

In the anti-saccades tests the stimulus moves, similarly to the visually guided saccades tests, to a random side of the screen and then back to its centre, repeatedly. However, in these tests the subject must direct their gaze towards the position of the screen opposite of the stimulus, and only look at the stimulus when it is in the centre of the screen (Fig. S2c). Each version of this test takes 36 s. Variables analyzed include latency, velocity and duration of reflexive saccades (saccades automatically performed towards the stimulus instead of away from it), anti-saccade latency and velocity, and accuracy; and number of anticipated, corrected and successful anti-saccades (see Fig. S3b, and Supporting Information).

In the smooth pursuit tests the stimulus moves across the screen with a constant period of 8 s (see Fig. S2d). The subject must follow the stimulus all time. The test lasts 32 s. In this case, a third version of the test was performed, where the stimulus moves horizontally with the same frequency, but its velocity increases and decreases at a constant rate. Latency, number of blinks, catch-up and back-up saccades (saccadic movements performed to go back to the position of the stimulus or keep up with its movement, respectively), square wave jerks (a specific kind of fixation instability), pursuit time, gain, error pursuit, and velocity error are measured in these tests.

In the Fixation test the subject is inquired to look at the immobile stimulus, showed in the centre of the screen (Fig. S2e). This test lasts 20 s. This test measures variables like the number of blinks, the bivariate contour ellipse area (BCEA) and the amplitude, duration, frequency and/or velocity of various kinds of fixation instabilities, which include saccades, micro-saccades, drifts and square-wave jerks.

For many variables, where the final result is averaged from the numerous eye movements performed during the test, a second variable is also generated, which provides information about the standard deviation of the original variable from which it is derived (i.e., latency and standard deviation of latency). A detailed definition of all variables measured in all tests is shown in the Supporting Information.

### Statistical analyses

All values are given as mean ± SEM unless otherwise stated. Results were analyzed using one of three options: one-way ANOVA followed by post-hoc Tukey’s multiple comparison test for variables both parametric and homoscedastic, Welch’s ANOVA followed by Games-Howell’s multiple comparison test for variables parametric but not homoscedastic, and Kruskal–Wallis’ test followed by Dunn’s test for non-parametric variables. Due to the number of variables analyzed in this study, *P* values obtained were corrected using the false discovery rate (FDR) method^41^. FDR values < 0.05 were considered significant. When analysing the correlation between different variables, Spearman’s correlation test was performed. For all statistical analyses, data were processed and analyzed using the software R version 4.1.1^42^.

Receiver operating characteristic (ROC) curves were performed using SPSS software (version 24.0; SPSS, Inc., Chicago, IL, USA), and two-sided *P* values < 0.05 were considered significant.

### Compliance with ethical standards

This study was performed in line with the principles of the Declaration of Helsinki. Study protocols were approved by the Scientific and Ethical Committees (No. 2017/291) of Hospitals Clinico and Arnau de Vilanova, Valencia, Spain. All participants were included after written informed consent.

## Supplementary Information

Supplementary Information.

## Data Availability

All data generated or analysed during this study are included in this published article and its Supporting Information file.
