# Supplementary material for: Minimal hepatic encephalopathy is associated to alterations in eye movements
Source: Sci Rep. 2022 Oct 7;12:16837. doi: 10.1038/s41598-022-21230-3 (PMC9547018; doi:10.1038/s41598-022-21230-3)
Supplement: Supplementary file 1 — Supplementary Information. [file 41598_2022_21230_MOESM1_ESM.docx]

**SUPPLEMENTARY INFORMATION**

**Minimal hepatic encephalopathy is associated to alterations in eye movements**

Franc Casanova-Ferrer, Cecilia E. García-Cena, Juan-Jose Gallego, Alessandra Fiorillo, Amparo Urios, Alberto Calvo-Córdoba, Maria-Pilar Ballester, María Pilar Ríos, Lucía Durbán, Marta R. Hidalgo, Francisco García, Vicente Felipo, Carmina Montoliu^*^

**Contents:**

**SUPPLEMENTARY METHODS**

- Description of Video-Oculography System
- Definition of variables measured in eye movement tests
- Supplementary References

**SUPPLEMENTARYFIGURES**

- **Figure S1.** OSCANN desk100 device
- **Figure S2.** Battery of eye movement tasks performed
- **Figure S3.** Main variables obtained after performing Visual Guided Saccades test, and Antisaccades test.

**SUPPLEMENTARY TABLES**

- **Table S1.** Technical characterization of the measure
- **Table S2.** Results of eye movement tests in the three groups of study: Horizontal and vertical visually-guided saccades
- **Table S3.**Results of eye movement tests in the three groups of study. Horizontal and vertical memory-guided saccades
- **Table S4.** Correlations of parameters of Fixation test with psychometric tests
- **Table S5.**Correlations of parameters of memory-guided saccades test with psychometric tests
- **Table S6.** Correlations of parameters of smooth pursuit test with psychometric tests
- **Table S7.**Correlations of parameters of sinusoidal smooth pursuit test with psychometric tests
- **Table S8.** Correlations of parameters of visually-guided saccades test with psychometric tests

**SUPPLEMENTARY METHODS**

**Description of video-oculography system**

## Hardware Description

OSCANN desk100 is a novel gaze-tracker designed for clinical practice use. It is based on VOG technology and the IR-camera captures images at 100 FPS. The measure is made over the dominant eye. Before the ocular movement test, the operator must check the dominance of the eyes and to move the camera in front of it.

In order to minimize the head movement, the camera and infrared light system were settled on mechanism attached to a chinrest. This mechanism was designed with 3 degree of freedom that allows adapting manually the system to the subject anatomy.

In FigureS1, a picture of the real system is shown. Two conventional screens with 22” and 120 Hz refreshing rate complete the system. The volunteer is settled in front of the screen at 60 cm from distance and the view field is free due to a hot mirror located in from of his/her eyes that filter the IR light. As part of the protocol, the size of the stimulus, its color and the back of the screen was fixed.

The technical characterization of the measure is presented in Table S1. The reader should note these values considered the human factor in the measure and the characterization included people in different ages, nationalities, with contact lenses as well as intraocular lenses. The values of precision, resolution and accuracy turn OSCANN desk100 system suitable for medical application.

**Table S1.** Technical characterization of the measure

| Quantity | Value SI |
| --- | --- |
| Measure limits | Horizontal: 40º; Vertical: 24º |
| Time resolution | 10 ms |
| Precision | Horizontal: 0.03º; Vertical: 0.03º |
| Accuracy | Horizontal: 0.4º; Vertical: 0.4º |
| Resolution | 0.0033º (RMS 0.0604º) |

Since the regulation point of view, OSCANN desk100 is a certified medical device under the international regulation and can be considered as a “safe device”.

**Definition of variables measured in eye movement tests**

***Visual guided saccades test***

Figures S2a and S3a describe the visual guided saccades test and parameters measured, respectively.

*Temporal parameters:*

- *Latency (ms):* Time elapsed between the change of the stimulus and the first ocular movement performed by the volunteer in response to the stimulus change.In the measure of latency (ms), the anticipated saccades (before 130 ms) were discarded.
- *Gain:* Relationship between the amplitude of the stimulus and that of ocular saccadic movement (gaze amplitude)
- *Peak of velocity (º/ms):*Maximum speed of the gaze in the saccadic movement

*Temporal parameters of return:*

- *Return Latency (ms)*: Elapsed time to initiate the movement to return to the center
- *Return Peak of velocity (º/ms):* Maximum speed of the gaze in the saccadic movement of returning to the center.

*Accuracy parameters*

- *Gaze Error (º):* Difference between the establishment of the gaze and the final position of the stimulus.If Error > 0 the saccade is hypermetria and if Error < 0 is hypometria.

*Counts*

- *Blinks*: Number of blinks
- *Anticipation*: Number of early visual saccades during the test

***Memory Guided Saccades Test***

Figure S2b describes the memory guided saccades test. In this test we measured the same variables than visual guided saccades plus the number and rate of correct memory saccades done by the subject. The accuracy variable was measured in the memory saccade.

***Antisaccades test***

Figures S2c and S3b describe the antisaccades test and parameters measured, respectively.

*Definitions*:

An antisaccade is correct if the volunteer performs a saccade movement in the opposite direction to the stimulus. When the volunteer performs a movement in the direction to the stimulus and then correct the gaze to the opposite side, the saccade is called “reflexive”.

Second-order reflexive anti-saccades are movements towards the stimulus, which cross the center of the screen, performed from a previous antisaccadic movement. This type of saccades is usually produced after a reflexive saccade.

*Temporal parameters:*

- *Latency of correct anti-saccade (ms):* Time between the stimulus change and the first anti-saccadic movement, during the correct visual anti-saccade.
- *Latency of reflexive anti-saccade (ms)*:Time between the stimulus change and the first anti-saccadic eye movement that crosses the center of the monitor during a reflexive visual anti-saccade.
- *Duration of the reflexive saccade (ms):* Fixation time during the reflexive saccade, that is, time between the first point of fixation of the reflexive saccade and the first point of fixation of the anti-saccade that crosses the center of the monitor.

*Temporal parameters of return:*

- *Return Latency (ms)*: Elapsed time to initiate the movement to return to the center
- *Return Peak of velocity (º/ms):* Maximum speed of the gaze in the saccadic movement of returning to the center.

*Accuracy parameters*

- *Gaze Error (º)*: Difference between the establishment of the gaze and the final position of the stimulus. If Error > 0 the saccade is hypermetria and if Error < 0 is hypometria.

*Counts*

- *Blinks*: Number of blinks
- *Blink frequency*: Frequency of blinks during the test
- *Anticipation*: Number of early visual saccades during the test
- *Correct anti-saccades*: Number of times that an anti-saccade is made without the presence of a pro-saccade during a visual anti-saccade during the test
- *Reflexive anti-saccades*: Number of times that a reflexive saccade is made during the test.
- *Second-order reflexive anti-saccades*: Number of times that second-order reflexive anti-saccades are made.
- *Incorrect anti-saccades*: Number of incorrect saccades

*Rates*

- *Success rate:* Number of times that correct anti-saccades have been made related to the total anti-saccades.
- *Rate of Corrected saccades:* Number of times that reflexive saccades have been made related to the total anti-saccades.
- *Rate of incorrect saccades:* Number of times that incorrect saccades have been made related to the total anti-saccades
- *Second-order reflexive anti-saccades rate*: Number of times that second-order reflexive anti-saccades have been made related to the total anti-saccades
- *Rate of anticipated saccades:* Number of times that anticipated saccades have been made related to the total anti-saccades.

***Smooth Pursuit Test***

Figure S2d shows the paradigm of the smooth pursuit test.

*Definitions*:

- *Catch-up Saccade* is a saccadic movement made in the sense of stimulus. It happens when the gaze remains behind the stimulus therefore this movement allows to reach it [1].
- *Back-up Saccade* is a saccadic movement made in the opposite sense of stimulus. It happens when the stimulus remains behind the gaze therefore this movement allows to reach it [1].
- *Square Mean Error* is the difference between the position of the stimulus and the gaze.

*Temporal parameters:*

- *Latency (ms)*: Time elapsed since starting the smooth pursuit test until the first ocular movement performed by the volunteer in response to the stimulus change.
- *Gain*: Measure of the speed matching, being the gain >1 when the eye anticipates to the stimulus movement, and the gain <1 when the eye is delayed.
- *Error pursuit*: Square Mean Error of the position including only the smooth pursuit performed during the test.
- *Error pursuit & saccades*
- *Velocity error*: Square Mean Error of velocity including only the smooth pursuit performed during the test.

*Counts*

- *Blinks*: Number of blinks
- *Catch-up*: Number of Catch-up saccades
- *Back-up*: Number of Back-up saccades
- *Square Wave Jerks*: Number of Square Wave Jerks

*Rates*

- *Pursuit time* (%): Percentage in which smooth pursuit is performed. This parameter measures the capacity to perform the test.

***Fixation test***

Figure S2e describes the Fixation test. *Definitions*:

- *Microsaccade* is an eye movement characterized by amplitude value of between 0.03 and 1 degrees and a duration value of 10 to 30 milliseconds [2].
- A *drift* is a slow movement that occurs when the gaze is fixed in a stimulus and it is placed between two microsaccades [3]. These particular movements are characterized by the duration (between 0.3 to 0.8 sec.) and by their amplitude (around 0.52º) [3].
- *Square Wave Jerks* (SWJ) are defined by two opposite saccadic movements whose amplitude are between 0.2º to 5º [4].
- *The Bivariate Contour Ellipse Area* (BCEA) is defined by the 69% of the fixation points. This variable represents the precision of the fixation during the test.

*Parameters for fixation accuracy*

- *Bivariate Contour Ellipse Area (BCEA) (ᴼ)^2^*
- *OX (º)*:Horizontal standard deviation of fixation points of the gaze
- *OY (º)*: Vertical standard deviation of fixation points of the gaze
- *Centroid X (º)*: Symmetry Center of BCEA in horizontal
- *Centroid Y (º)*: Symmetry Center of BCEA in vertical

*Counts*

- *Blinks*
- *Saccades*
- *Microsaccades*
- *Drift*
- *Monophasic and biphasicSquare Wave Jerks* (SWJ)
- *Distractions*

*Parameters of Microsaccades*

- *Amplitude (º):*mean of the amplitudes of microsaccades performed during the test
- *Velocity (º/s):* Mean of velocity of microsaccades performed during the test
- *Peak of Velocity (º/s):* Mean of peak velocity of microsaccades performed during the test.
- *Frequency*

*Parameters of Drift*

- *Amplitude (º):* mean of the amplitudes of drift performed during the test
- *Velocity (º/s):* Mean of velocity of drift performed during the test
- *Peak of Velocity (º/s):* Mean of peak velocity of drift performed during the test.

*Parameters of SWJ:*

- *Amplitude (º):* Mean of the amplitudes of SWJ performed during the test
- *Time (ms):* Mean of time of SWJ performed during the test

**Supplementary References**

1. Flechtner KM, Steinacher, Sauer R, Mackert A. Smooth pursuit eye movements of patients with schizophrenia and affective disorder during clinical treatment. Eur Arch Psychiatry Clin Neurosci. 2002; 252:49-53.
2. Macknik SL, Martinez-Conde S, Hubel DH. The Role of Fixational Eye Movements in Visual Perception. Nature Rev Neurosci 2004; 5:229-240.
3. Martinez-Conde S, Macknik SL, Troncos XG, Hubel DH. Microsaccades: a neurophysiological analysis. Trends in Neurosciences. 2009; 32(9):463-475
4. Mario Bettenb, Potsdam Cognitive and Science Series. Universitatsverlag Potsdam. ISBN: 9783869561226.

**SUPPLEMENTARY FIGURES**


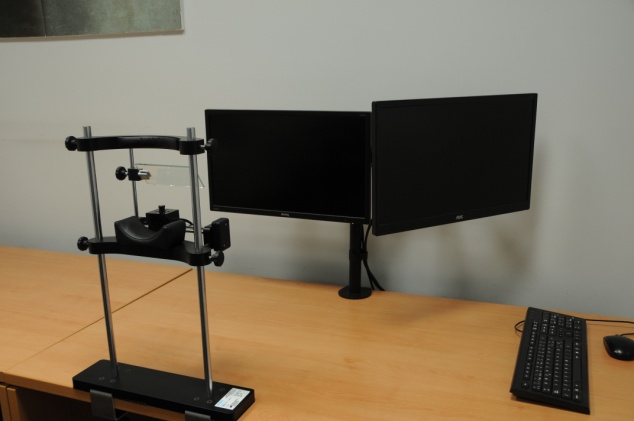


**Figure S1.** OSCANN desk100 device.


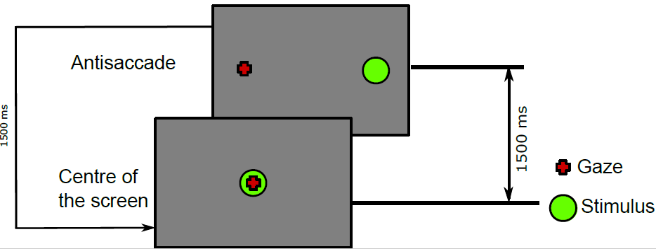


**c**

**Antisaccades Test**


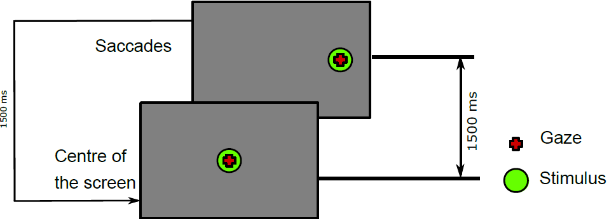


**a**

**Visual Guided Saccades Test**

**b**

**Memory Guided Saccades Test**


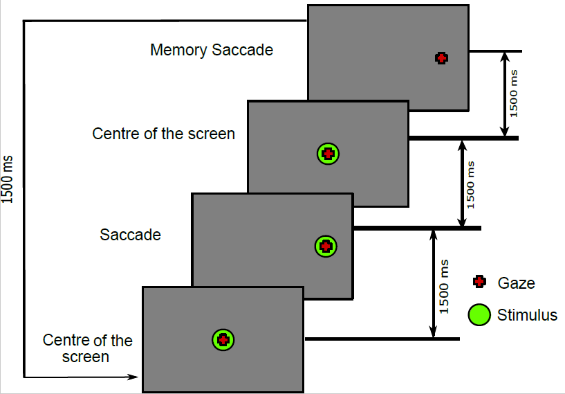


**d**

**Smooth Pursuit Test**


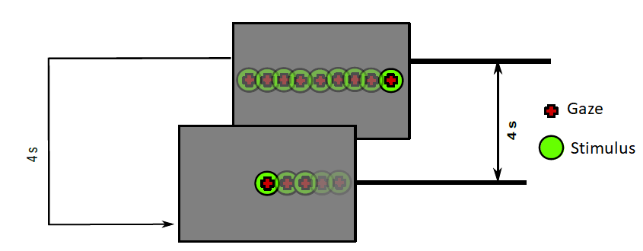


**Fixation Test**


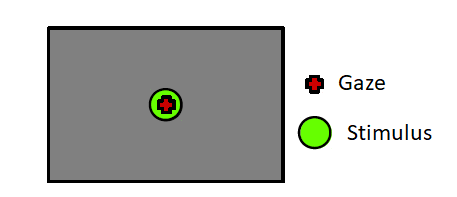


**e**

**Figure S2.** Battery of eye movement tasks performed. **(a)**Visual Guided Saccades Test. **(b)** Memory Guided Saccades Test. **(c)** Antisaccades test. (**d)** Smooth Pursuit test. **(e)** Fixation test.

**a**


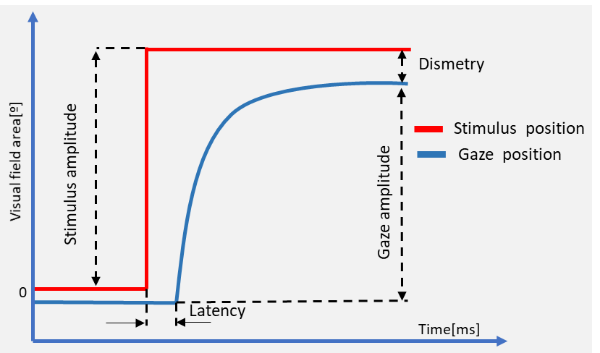


**b**


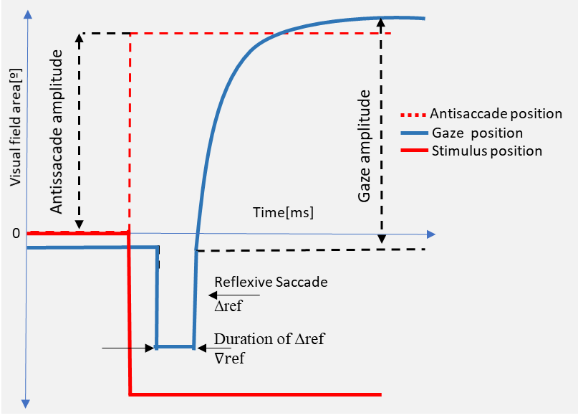


**Figure S3.** Main variables obtained after performing Visual Guided Saccades Test **(a)**, and Antisaccades test **(b)**

**SUPPLEMENTARY TABLES**

**Table S2.** Results of eye movement tests in the three groups of study: Horizontal and vertical visual guided saccades

|  | **Controls** | **NMHE patients P vs. control** | **MHE patients P vs. control** | **MHE patients P vs. NMHE** | **Global FDR values** |
| --- | --- | --- | --- | --- | --- |
| **HORIZONTAL VISUAL GUIDED SACCADES** | |  |  |  |  |
| Latency (ms)^c^ | 227 ± 3.9 | 230 ± 3.3 | 258 ± 9.8* | 0.014 | ns |
| Standard deviation of latency^c^ | 39 ± 3.9 | 39 ± 1.9 | 62 ± 6.5** | 0.001 | 0.03 |
| Gain^c^ | 1 ± 0.01 | 1 ± 0.01 | 1.1 ± 0.01 | ns | ns |
| Standard deviation of gain^c^ | 0.08 ± 0.01 | 0.09 ± 0.01 | 0.1 ± 0.01 | ns | ns |
| Peak of velocity (º/ms)^c^ | 374 ± 18 | 341 ± 11 | 338 ± 17 | ns | ns |
| Standard deviation of peak of velocity^c^ | 154 ± 8.9 | 142 ± 4.3 | 137 ± 8 | ns | ns |
| Positive error^c^ | 0.68 ± 0.06 | 0.71 ± 0.07 | 0.88 ± 0.11 | ns | ns |
| Standard deviation of positive error^c^ | 0.38 ± 0.03 | 0.44 ± 0.04 | 0.64 ± 0.13 | ns | ns |
| Negative error^c^ | -0.52 ± 0.13 | -0.48 ± 0.06 | -0.52 ± 0.12 | ns | ns |
| Standard deviation of negative error^c^ | 0.47 ± 0.14 | 0.32 ± 0.05 | 0.32 ± 0.13 | ns | ns |
| Blinks^c^ | 3.6 ± 0.64 | 2.9 ± 0.45 | 4.2 ± 0.88 | ns | ns |
| Number of anticipated saccades^c^ | 0.36 ± 0.1 | 0.56 ± 0.1 | 0.92 ± 0.24 | ns | ns |
| Returnlatency (ms)^c^ | 209 ± 5 | 214 ± 2.7 | 230 ± 7.4* | ns | ns |
| Standard deviation of return latency^c^ | 35 ± 3.4 | 38 ± 2.2 | 44 ± 4.5 | ns | ns |
| Return peak of velocity (º/ms)^c^ | 380 ± 21 | 332 ± 8.2 | 317 ± 15 | ns | ns |
| Standard deviation of return peak of velocity^c^ | 180 ± 10 | 173 ± 6.7 | 163 ± 9.5 | ns | ns |
| **VERTICAL VISUAL GUIDED SACCADES** | |  |  |  |  |
| Latency (ms)^c^ | 246 ± 5.2 | 258 ± 4.2 | 274 ± 8.8* | ns | ns |
| Standard deviation of latency^c^ | 46 ± 3.5 | 45 ± 2.6 | 46 ± 3.4 | ns | ns |
| Gain^c^ | 1 ± 0.01 | 1 ± 0.01 | 1 ± 0.02 | ns | ns |
| Standard deviation of gain^c^ | 0.11 ± 0.01 | 0.14 ± 0.01 | 0.13 ± 0.02 | ns | ns |
| Peak of velocity (º/ms)^c^ | 282 ± 11 | 258 ± 6.8 | 240 ± 12* | ns | ns |
| Standard deviation of peak of velocity ^b^ | 86 ± 5.3 | 95 ± 4.5 | 92 ± 5.2 | ns | ns |
| Positive error^c^ | 0.65 ± 0.06 | 0.75 ± 0.08 | 0.74 ± 0.12 | ns | ns |
| Standard deviation of positive error^c^ | 0.41 ± 0.06 | 0.35 ± 0.03 | 0.27 ± 0.03 | ns | ns |
| Negative error^c^ | -0.37 ± 0.05 | -0.43 ± 0.05 | -0.45 ± 0.09 | ns | ns |
| Standard deviation of negative error^c^ | 0.35 ± 0.07 | 0.25 ± 0.03 | 0.22 ± 0.05 | ns | ns |
| Blinks^c^ | 3.1 ± 0.53 | 2.8 ± 0.4 | 3.4 ± 0.57 | ns | ns |
| Anticipatedsaccades^c^ | 0.58 ± 0.23 | 0.7 ± 0.13 | 0.4 ± 0.13 | ns | ns |
| Returnlatency (ms) ^a^ | 218 ± 5.9 | 238 ± 3.5* | 263 ± 8.8*** | 0.007 | <0.001 |
| Standard deviation of return latency^c^ | 30 ± 2.4 | 44 ± 3.1** | 49 ± 6** | ns | ns |
| Return peak of velocity (º/ms) ^a^ | 273 ± 11 | 251 ± 7.4 | 232 ± 14 | ns | ns |
| Standard deviation of return peak of velocity^c^ | 93 ± 5.8 | 97 ± 3.9 | 93 ± 8.2 | ns | ns |

All values are expressed as mean±SEM. Abbreviations: NMHE and MHE, patients without and with minimal hepatic encephalopathy according to PHES. Differences between groups were analysed using one of three possibilities: one-way ANOVA followed by Tukey´s multiple comparison test (a) parametric and homoscedastic variables, Welch’s ANOVA followed by Games-Howell’s multiple comparison test (b) for parametric but non-homoscedastic variables, and Kruskal-Wallis followed by Dunn’s multiple comparison test (c) for non-parametric variables. Significant differences compared to controls are indicated by asterisks: *p<0.05; **p<0.01; ***p<0.001; ns, non-significant. Detailed definitions of eye movement variables are provided above.

**Table S3.** Results of eye movement tests in the three groups of study. Horizontal and vertical memory-guided saccades.

|  | **Controls** | **NMHE patients P vs. control** | **MHE patients P vs. control** | **MHE patients P vs. NMHE** | **Global FDR values** |
| --- | --- | --- | --- | --- | --- |
| **HORIZONTAL MEMORY-GUIDED SACCADES** | |  |  |  |  |
| Latency (ms)^c^ | 294 ± 11 | 352 ± 12** | 393 ± 23** | ns | 0.021 |
| Standard deviation of latency^c^ | 93 ± 10 | 135 ± 9.4* | 121 ± 13 | ns | ns |
| Gain^c^ | 1 ± 0.02 | 1 ± 0.02 | 0.93 ± 0.03* | 0.040 | ns |
| Standard deviation of gain^c^ | 0.17 ± 0.02 | 0.21 ± 0.02 | 0.21 ± 0.04 | ns | ns |
| Peak of velocity (º/ms)^c^ | 358 ± 17 | 335 ± 15 | 299 ± 25 | ns | ns |
| Standard deviation of peak of velocity^c^ | 150 ± 12 | 147 ± 8.8 | 118 ± 12 | ns | ns |
| Positive error^c^ | 1.7 ± 0.21 | 1.8 ± 0.15 | 1.2 ± 0.13 | ns | ns |
| Standard deviation of positive error^c^ | 0.91 ± 0.1 | 1.2 ± 0.13 | 0.69 ± 0.08 | ns | ns |
| Negative error^c^ | -1.1 ± 0.18 | -1.3 ± 0.14 | -1.5 ± 0.28 | ns | ns |
| Standard deviation of negative error^c^ | 0.7 ± 0.14 | 0.84 ± 0.11 | 1 ± 0.19 | ns | ns |
| Blinks^c^ | 7.4 ± 1.3 | 7.4 ± 0.95 | 11 ± 2.3 | ns | ns |
| Number of anticipated saccades^c^ | 3.4 ± 0.48 | 2.4 ± 0.29 | 1.9 ± 0.35 | ns | ns |
| Number of correct saccades^c^ | 10 ± 0.48 | 8.6 ± 0.47 | 5.1 ± 0.66*** | <0.001 | <0.001 |
| Returnlatency (ms)^c^ | 291 ± 16 | 327 ± 13 | 325 ± 22 | ns | ns |
| Standard deviation of return latency^c^ | 90 ± 8.9 | 110 ± 7.7 | 102 ± 14 | ns | ns |
| Return peak of velocity (º/ms) ^a^ | 392 ± 25 | 346 ± 13 | 310 ± 27 | ns | ns |
| Standard deviation of return peak of velocity ^a^ | 171 ± 12 | 165 ± 7.4 | 133 ± 16 | ns | ns |
| **VERTICAL MEMORY-GUIDED SACCADES** | |  |  |  |  |
| Latency (ms)^c^ | 267 ± 16 | 337 ± 15** | 452 ± 43*** | 0.009 | 0.001 |
| Standard deviation of latency^c^ | 75 ± 14 | 122 ± 12* | 168 ± 33* | ns | ns |
| Gain ^a^ | 1 ± 0.02 | 0.97 ± 0.02 | 1 ± 0.05 | ns | ns |
| Standard deviation of gain^c^ | 0.16 ± 0.02 | 0.19 ± 0.01 | 0.28 ± 0.05 | ns | ns |
| Peak of velocity (º/ms) ^b^ | 259 ± 11 | 240 ± 12 | 230 ± 28 | ns | ns |
| Standard deviation of peak of velocity^c^ | 96 ± 7.8 | 100 ± 5.6 | 90 ± 22 | ns | ns |
| Positive error^c^ | 1.4 ± 0.14 | 1.2 ± 0.11 | 2 ± 0.24 | 0.016 | ns |
| Standard deviation of positive error^c^ | 0.8 ± 0.09 | 0.77 ± 0.08 | 1.4 ± 0.23 | ns | ns |
| Negative error^c^ | -0.85 ± 0.13 | -1.3 ± 0.12* | -1.3 ± 0.31 | ns | ns |
| Standard deviation of negative error^c^ | 0.47 ± 0.1 | 0.72 ± 0.08 | 0.76 ± 0.35 | ns | ns |
| Blinks^c^ | 5.1 ± 0.82 | 7 ± 0.94 | 8.5 ± 1.5 | ns | ns |
| Number of anticipated saccades^c^ | 2.5 ± 0.4 | 2.2 ± 0.28 | 1.5 ± 0.26 | ns | ns |
| Number of correct saccades^c^ | 6.9 ± 0.39 | 5.8 ± 0.33* | 2.7 ± 0.48*** | <0.001 | <0.001 |
| Returnlatency (ms)^c^ | 249 ± 12 | 309 ± 15** | 327 ± 27* | ns | ns |
| Standard deviation of return latency^c^ | 78 ± 7.8 | 96 ± 11 | 102 ± 19 | ns | ns |
| Return peak of velocity (º/ms) ^a^ | 259 ± 13 | 235 ± 10 | 219 ± 26 | ns | ns |
| Standard deviation of return peak of velocity^c^ | 115 ± 11 | 96 ± 4.3 | 112 ± 19 | ns | ns |

All values are expressed as mean±SEM. Abbreviations: NMHE and MHE, patients without and with minimal hepatic encephalopathy according to PHES. Differences between groups were analysed using one of three possibilities: one-way ANOVA followed by Tukey´s multiple comparison test (a) parametric and homoscedastic variables, Welch’s ANOVA followed by Games-Howell’s multiple comparison test (b) for parametric but non-homoscedastic variables, and Kruskal-Wallis followed by Dunn’s multiple comparison test (c) for non-parametric variables. Significant differences compared to controls are indicated by asterisks: *p<0.05; **p<0.01; ***p<0.001; ns, non-significant. Detailed definitions of eye movement variables are provided above.

**Table S4.**Correlations of parameters of Fixation test with psychometric tests

| **FIXATION TEST** | | | | | | | | | | | | | | | | | | | | | |  |
| --- | --- | --- | --- | --- | --- | --- | --- | --- | --- | --- | --- | --- | --- | --- | --- | --- | --- | --- | --- | --- | --- | --- |
|  |  | Blinks | | Number of saccades | | Number of distractions | | BCEA (º) | | OX | | OY | | Centroid X | | Micros.  Amplitude | | Velocity microsaccades | | Peak of velocity of microsaccades | |  |
| PHES score | r | | -0.188 | | -0.256 | | -0.198 | | -0.245 | | -0.284 | | -0.193 | | 0.0682 | | **-0.320** | | **-0.297** | | -0.274 | |
|  | p | 0.032 | | 0.004 | | 0.026 | | 0.006 | | 0.001 | | 0.030 | | ns | | **<0.001** | | **<0.001** | | 0.002 | |  |
| DST (items completed) | r | -0.179 | | -0.213 | | -0.224 | | **-0.347** | | **-0.322** | | **-0.347** | | 0.1348 | | -0.300 | | -0.276 | | -0.261 | |  |
|  | p | ns | | 0.024 | | 0.015 | | **<0.001** | | **<0.001** | | **<0.001** | | ns | | 0.001 | | 0.003 | | 0.005 | |  |
| NCT-A (seconds) | r | 0.153 | | 0.256 | | 0.161 | | **0.321** | | **0.323** | | 0.241 | | -0.038 | | **0.381** | | **0.337** | | **0.352** | |  |
|  | p | ns | | 0.006 | | ns | | **<0.001** | | **<0.001** | | 0.008 | | ns | | **<0.001** | | **<0.001** | | **<0.001** | |  |
| NCT-B (seconds) | r | 0.1634 | | **0.365** | | **0.310** | | 0.271 | | 0.290 | | 0.271 | | -0.186 | | 0.265 | | 0.229 | | 0.238 | |  |
|  | p | ns | | **<0.001** | | **<0.001** | | 0.004 | | 0.001 | | 0.003 | | 0.046 | | 0.005 | | 0.016 | | 0.012 | |  |
| SD (seconds) | r | 0.059 | | 0.1832 | | 0.1166 | | 0.282 | | **0.313** | | 0.222 | | -0.079 | | 0.246 | | 0.273 | | 0.249 | |  |
|  | p | ns | | ns | | ns | | 0.002 | | **<0.001** | | 0.017 | | ns | | 0.009 | | 0.003 | | 0.008 | |  |
| LTT (seconds + errors) | r | 0.147 | | 0.259 | | 0.1730 | | 0.275 | | 0.293 | | 0.292 | | 0.052 | | 0.282 | | 0.306 | | 0.271 | |  |
|  | p | ns | | 0.006 | | ns | | 0.003 | | 0.001 | | 0.001 | | ns | | 0.002 | | 0.001 | | 0.003 | |  |

Spearman correlation parameters are shown. PHES, Psychometric Hepatic Encephalopathy Score; DST, Digit Symbol Test; NCT-A, NCT-B: Number Connection Test A and B; SD, Serial Dotting Test; LTT, Line Tracing Test; ns, not significant. More significant correlations are highlighted in bold.

**Table S5.** Correlations of parameters of memory-guided saccades test with psychometric tests

|  |  | **HORIZONTAL MEMORY-GUIDED SACCADES** | | | | | | **VERTICAL MEMORY-GUIDED SACCADES** | | | |
| --- | --- | --- | --- | --- | --- | --- | --- | --- | --- | --- | --- |
|  |  | Latency | Gain | Peak of velocity | Negative error | Anticipated saccades | Correct saccades | Latency | Negative error | Correct saccades | Return latency |
| PHES score | r | **-0.346** | 0.173 | 0.253 | 0.320 | 0.219 | **0.434** | **-0.371** | 0.223 | **0.484** | -0.204 |
|  | p | **<0.001** | ns | 0.007 | 0.001 | 0.014 | **<0.001** | **<0.001** | ns | **<0.001** | 0.038 |
| DST (items completed) | r | **-0.426** | 0.159 | 0.2112 | 0.2485 | 0.1484 | **0.544** | **-0.375** | 0.294 | **0.549** | -0.133 |
|  | p | **<0.001** | ns | 0.033 | 0.021 | ns | **<0.001** | **<0.001** | 0.018 | **<0.001** | ns |
| NCT-A (seconds) | r | **0.357** | -0.112 | -0.197 | -0.398 | -0.204 | **-0.452** | **0.460** | -0.255 | **-0.460** | 0.243 |
|  | p | **<0.001** | ns | 0.045 | <0.001 | 0.029 | **<0.001** | **<0.001** | 0.039 | **<0.001** | 0.016 |
| NCT-B (seconds) | r | **0.354** | -0.136 | -0.235 | -0.276 | -0.168 | **-0.523** | **0.384** | -0.311 | **-0.472** | 0.169 |
|  | p | **<0.001** | ns | 0.018 | 0.011 | ns | **<0.001** | **<0.001** | 0.013 | **<0.001** | ns |
| SD (seconds) | r | **0.405** | -0.073 | -0.190 | -0.262 | -0.189 | -0.314 | 0.300 | -0.256 | -0.304 | 0.032 |
|  | p | **<0.001** | ns | ns | 0.015 | 0.045 | <0.001 | 0.003 | 0.040 | <0.001 | ns |
| LTT (seconds + errors) | r | 0.300 | -0.243 | -0.255 | -0.248 | -0.240 | **-0.487** | 0.282 | -0.157 | **-0.516** | 0.080 |
|  | p | 0.002 | 0.015 | 0.009 | 0.022 | 0.010 | **<0.001** | 0.006 | ns | **<0.001** | ns |

Spearman correlation parameters are shown. PHES, Psychometric Hepatic Encephalopathy Score; DST, Digit Symbol Test; NCT-A, NCT-B: Number Connection Test A and B; SD, Serial Dotting Test; LTT, Line Tracing Test; ns, not significant. More significant correlations are highlighted in bold.

**Table S6.** Correlations of parameters of smooth pursuit test with psychometric tests

|  |  | **HORIZONTAL SMOOTH PURSUIT** | | | | | | **VERTICAL SMOOTH PURSUIT** | | | | | | |
| --- | --- | --- | --- | --- | --- | --- | --- | --- | --- | --- | --- | --- | --- | --- |
|  |  | Back-up saccades | Square wave jerks | Pursuit time | Total mean squared error of position | Pursuit mean squared error of position | Gain | Catch-up saccades | Back-up saccades | Square wave jerks | Pursuit time | Total mean squared error of position | Pursuit mean squared error of position | Gain |
| PHES | r | **-0.301** | -0.233 | 0.207 | **-0.298** | **-0.295** | **0.343** | -0.219 | -0.217 | -0.267 | 0.185 | **-0.367** | **-0.370** | **0.370** |
|  | p | **<0.001** | 0.007 | 0.018 | **<0.001** | **<0.001** | **<0.001** | 0.013 | 0.014 | 0.002 | 0.042 | **<0.001** | **<0.001** | **<0.001** |
| DST (items completed) | r | -0.263 | -0.187 | 0.211 | **-0.370** | **-0.373** | **0.396** | -0.249 | -0.229 | **-0.340** | 0.199 | **-0.447** | **-0.422** | **0.432** |
|  | p | 0.003 | 0.042 | 0.023 | **<0.001** | **<0.001** | **<0.001** | 0.008 | 0.015 | **<0.001** | 0.039 | **<0.001** | **<0.001** | **<0.001** |
| NCT-A (seconds) | r | 0.251 | 0.273 | -0.263 | **0.394** | **0.398** | **-0.488** | 0.127 | 0.267 | **0.496** | -0.255 | **0.432** | **0.418** | **-0.430** |
|  | p | 0.005 | 0.002 | 0.003 | **<0.001** | **<0.001** | **<0.001** | ns | 0.004 | **<0.001** | 0.006 | **<0.001** | **<0.001** | **<0.001** |
| NCT-B (seconds) | r | 0.214 | 0.171 | -0.263 | **0.433** | **0.433** | **-0.487** | 0.216 | 0.297 | **0.386** | -0.262 | **0.507** | **0.494** | **-0.461** |
|  | p | 0.021 | ns | 0.004 | **<0.001** | **<0.001** | **<0.001** | 0.025 | 0.001 | **<0.001** | 0.007 | **<0.001** | **<0.001** | **<0.001** |
| SD (seconds) | r | 0.154 | 0.304 | -0.083 | **0.364** | **0.363** | -0.274 | 0.1641 | 0.180 | 0.306 | -0.044 | 0.287 | 0.269 | -0.282 |
|  | p | ns | <0.001 | ns | **<0.001** | **<0.001** | 0.003 | ns | ns | 0.001 | ns | 0.002 | 0.005 | 0.003 |
| LTT (seconds + errors) | r | 0.235 | 0.204 | -0.206 | 0.301 | 0.304 | **-0.393** | 0.250 | 0.193 | 0.262 | -0.214 | **0.422** | **0.417** | **-0.421** |
|  | p | 0.010 | 0.026 | 0.026 | 0.001 | <0.001 | **<0.001** | 0.008 | 0.043 | 0.005 | 0.026 | **<0.001** | **<0.001** | **<0.001** |

Spearman correlation parameters are shown. PHES, Psychometric Hepatic Encephalopathy Score; DST, Digit Symbol Test; NCT-A, NCT-B: Number Connection Test A and B; SD, Serial Dotting Test; LTT, Line Tracing Test; ns, not significant. More significant correlations are highlighted in bold.

**Table S7.** Correlations of parameters of sinusoidal smooth pursuit test with psychometric tests

|  |  | **SINUSOIDAL SMOOTH PURSUIT** | | | | | |
| --- | --- | --- | --- | --- | --- | --- | --- |
|  |  | Catch-up saccades | Pursuit time | Latency | Total mean squared error of position | Pursuit mean squared error of position | Gain |
| PHES | r | -0.215 | 0.199 | -0.316 | **-0.334** | **-0.330** | 0.303 |
|  | p | 0.024 | 0.044 | 0.001 | **<0.001** | **<0.001** | 0.002 |
| DST (items completed) | r | -0.313 | 0.285 | **-0.405** | **-0.398** | **-0.399** | 0.319 |
|  | p | 0.001 | 0.003 | **<0.001** | **<0.001** | **<0.001** | 0.001 |
| NCT-A (seconds) | r | 0.304 | -0.306 | **0.364** | **0.435** | **0.431** | **-0.400** |
|  | p | 0.001 | 0.001 | **<0.001** | **<0.001** | **<0.001** | **<0.001** |
| NCT-B (seconds) | r | 0.224 | **-0.341** | **0.348** | **0.496** | **0.489** | **-0.395** |
|  | p | 0.021 | **<0.001** | **<0.001** | **<0.001** | **<0.001** | **<0.001** |
| SD (seconds) | r | 0.1523 | -0.074 | 0.317 | 0.308 | 0.308 | -0.220 |
|  | p | ns | ns | 0.001 | 0.001 | 0.001 | 0.028 |
| LTT (seconds + errors) | r | 0.222 | -0.297 | **0.419** | **0.408** | **0.402** | -0.271 |
|  | p | 0.021 | 0.002 | **<0.001** | **<0.001** | **<0.001** | 0.006 |

Spearman correlation parameters are shown. PHES, Psychometric Hepatic Encephalopathy Score; DST, Digit Symbol Test; NCT-A, NCT-B: Number Connection Test A and B; SD, Serial Dotting Test; LTT, Line Tracing Test; ns, not significant. More significant correlations are highlighted in bold.

**Table S8.** Correlations of parameters of visual guided saccades test with psychometric tests

| **VISUAL GUIDED SACCADES** | | | | | | | |
| --- | --- | --- | --- | --- | --- | --- | --- |
|  |  | HORIZONTAL | | | | VERTICAL |  |
|  |  | Latency | Standard deviation of latency | Anticipated saccades | Return latency (ms) | Latency | Return latency (ms) |
| PHES | r | -0.292 | **-0.344** | -0.179 | -0.239 | **-0.297** | **-0.439** |
|  | p | 0.001 | **<0.001** | 0.043 | 0.008 | **<0.001** | **<0.001** |
| DST (items completed) | r | **-0.391** | **-0.400** | -0.155 | **-0.362** | **-0.478** | **-0.546** |
|  | p | **<0.001** | **<0.001** | ns | **<0.001** | **<0.001** | **<0.001** |
| NCT-A (seconds) | r | **0.319** | **0.483** | 0.169 | 0.300 | **0.393** | **0.457** |
|  | p | **<0.001** | **<0.001** | ns | 0.001 | **<0.001** | **<0.001** |
| NCT-B (seconds) | r | 0.244 | **0.370** | 0.221 | 0.196 | **0.337** | **0.481** |
|  | p | 0.014 | **<0.001** | 0.020 | 0.047 | **<0.001** | **<0.001** |
| SD (seconds) | r | 0.263 | **0.328** | 0.108 | 0.217 | **0.318** | **0.341** |
|  | p | 0.006 | **<0.001** | ns | 0.025 | **<0.001** | **<0.001** |
| LTT (seconds + errors) | r | 0.298 | **0.341** | 0.191 | 0.251 | 0.309 | **0.425** |
|  | p | 0.001 | **<0.001** | 0.043 | 0.009 | 0.001 | **<0.001** |

Spearman correlation parameters are shown. PHES, Psychometric Hepatic Encephalopathy Score; DST, Digit Symbol Test; NCT-A, NCT-B: Number Connection Test A and B; SD, Serial Dotting Test; LTT, Line Tracing Test; ns, not significant. More significant correlations are highlighted in bold.
